# Chemical signaling in the developing avian retina: Focus on cyclic AMP and AKT-dependent pathways

**DOI:** 10.3389/fcell.2022.1058925

**Published:** 2022-12-09

**Authors:** A. T. Duarte-Silva, L. G. R. Ximenes, M. Guimarães-Souza, I. Domith, R. Paes-de-Carvalho

**Affiliations:** ^1^ Program of Neurosciences, Institute of Biology, Fluminense Federal University, Niterói, Brazil; ^2^ Department of Neurobiology, Institute of Biology, Fluminense Federal University, Niterói, Brazil

**Keywords:** cyclic AMP, calcium, adenosine, dopamine, glutamate, nitric oxide, vitamin C, Akt

## Abstract

Communication between developing progenitor cells as well as differentiated neurons and glial cells in the nervous system is made through direct cell contacts and chemical signaling mediated by different molecules. Several of these substances are synthesized and released by developing cells and play roles since early stages of Central Nervous System development. The chicken retina is a very suitable model for neurochemical studies, including the study of regulation of signaling pathways during development. Among advantages of the model are its very well-known histogenesis, the presence of most neurotransmitter systems found in the brain and the possibility to make cultures of neurons and/or glial cells where many neurochemical functions develop in a similar way than in the intact embryonic tissue. In the chicken retina, some neurotransmitters or neuromodulators as dopamine, adenosine, and others are coupled to cyclic AMP production or adenylyl cyclase inhibition since early stages of development. Other substances as vitamin C and nitric oxide are linked to the major neurotransmitter glutamate and AKT metabolism. All these different systems regulate signaling pathways, including PKA, PKG, SRC, AKT and ERK, and the activation of the transcription factor CREB. Dopamine and adenosine stimulate cAMP accumulation in the chick embryo retina through activation of D1 and A2a receptors, respectively, but the onset of dopamine stimulation is much earlier than that of adenosine. However, adenosine can inhibit adenylyl cyclase and modulate dopamine-dependent cAMP increase since early developmental stages through A1 receptors. Dopamine stimulates different PKA as well as EPAC downstream pathways both in intact tissue and in culture as the CSK-SRC pathway modulating glutamate NMDA receptors as well as vitamin C release and CREB phosphorylation. By the other hand, glutamate modulates nitric oxide production and AKT activation in cultured retinal cells and this pathway controls neuronal survival in retina. Glutamate and adenosine stimulate the release of vitamin C and this vitamin regulates the transport of glutamate, activation of NMDA receptors and AKT phosphorylation in cultured retinal cells. In the present review we will focus on these reciprocal interactions between neurotransmitters or neuromodulators and different signaling pathways during retinal development.

## 1 Introduction

### 1.1 The chicken retina as a model for studies of central nervous system neurochemical development

**FIGURE 1:**
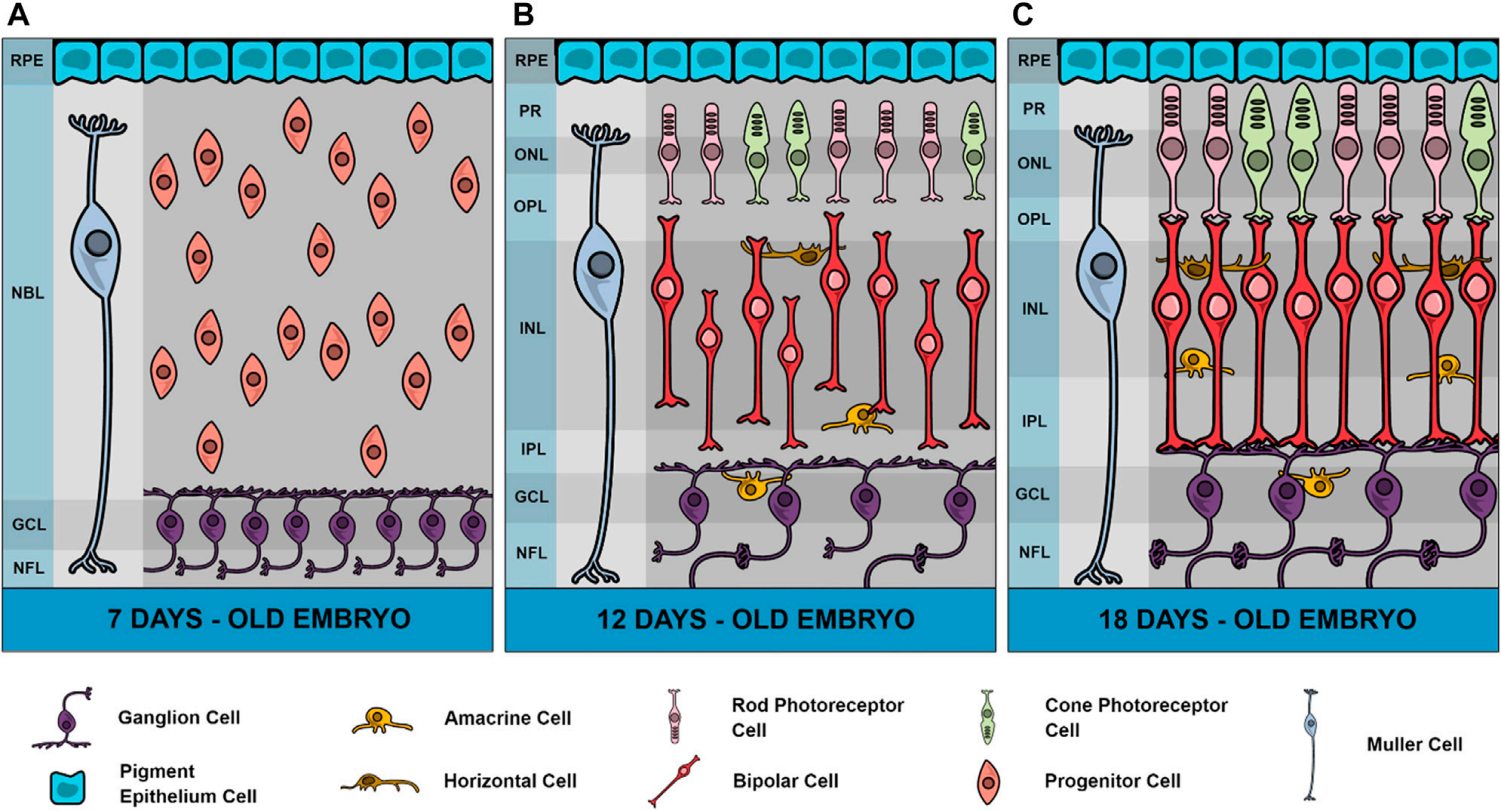
Schematic representation of chick retina in 3 different developmental stages. In embryonic day 7 (E7), neuroblasts are still dividing and a high number of ganglion cells are fully differentiated in a ganglion cell layer (GCL) and sending processes through a growing nerve fiber layer (NFL). Plexiform layers are not yet defined and instead is observed an extensive neuroblastic layer (NBL) and the retinal pigmented epithelium (RPE). Muller glial cells are also present in this stage. In E12 retinas, both inner and outer plexiform layers (IPL and OPL, respectively) are observed where neuronal processes progressively grow but synaptic contacts are observed only after E13 in IPL. Amacrine cells can be observed in both inner nuclear layer (INL) and GCL (displaced amacrine cells) and horizontal cells in the external limit of INL. From E12 to E18 there is a progressive increase of IPL and OPL thickness. Photoreceptors present a late development in the chick retina with the external segment (PR) beginning to grow in E15 and synapses between photoreceptors and bipolar cells appearing only in E17. In E19, the retina can be considered mature, and the first light responses are observed. Variations in thickness of retinal layers were drawn according to Zareen et al. (2010), but total retina thickness in different stages are not represented in this figure.

The retina is a part of the Central Nervous System (CNS) involved in receiving, transducing, and modulating the light signals from the environment. The chicken retina is a very suitable model system for the study of CNS development since it can be easily obtained during most part of embryonic development and its histogenesis is relatively well known (Adler, 2000). Although few classes of cells are anatomically discernible, the chick retina differentiates more than a hundred cell types, as recently described using single cell transcriptomics, distributed among the six classes conserved across vertebrates (Yamagata et al., 2021), including photoreceptors, bipolar and ganglion cells comprising the vertical pathway, and horizontal and amacrine cells responsible for the horizontal modulation of neuronal activity. Moreover, a major glial cell type is the Muller cell which makes contact with neurons in the almost complete retinal extension (reviewed by Reichenbach and Bringmann, 2013). The chick retina is also a good model for studying nervous system development since it presents many of the neurotransmitters and neuromodulators present in the CNS (Calaza and Gardino, 2010). Figure 1 shows the general chick retina structure in three major periods of development, 7-day-old embryo (E7), E12 and E18. As can be observed, one important characteristic is the increase of plexiform layers where synaptic contacts, neurotransmitters and receptors are predominantly localized.

### 1.2 The chicken retina as a model to study retinal degenerations

Many studies discuss the advantages of using avian retina in eye disease models (Wisely et al., 2017). Studies show the use of the chick retina as a model in corneal diseases because of its anatomical and molecular similarities with the retina of humans (Fowler et al., 2004; Martínez-García et al., 2006; Mangioris et al., 2011). Many retinal diseases are related to dysfunctions in the glutamatergic system such as diabetic retinopathy, glaucoma and retinal ischemia (reviewed by Ishikawa, 2013). Retinal detachment causes changes in photoreceptors, which can degenerate and undergo apoptosis, and in Muller’s glia, which proliferate and hypertrophy, contributing to the destructive scarring. Microglia can become reactive and accumulate in the retina. These events cause changes in the outer nuclear layer of the retina. Study with chicks with postnatal age between 7 and 21 days shows the advantage of this model in studies of retinal detachment (Cebulla et al., 2012). Chicken embryos were also used for studies of diabetic retinopathy in which the embryos were exposed to high concentrations of glucose on the first day of development to cause hyperglycemia and this caused damage to the embryo’s eye development and the molecular mechanism of this malformation was studied demonstrating the advantages of this model (Zhang et al., 2016). In addition, there are also reports of the use of chicken embryos for retinoblastoma studies (Busch et al., 2015; Nair et al., 2022), myopia and hyperopia (Wildsoet and Schmid, 2000; reviewed by Iribarren, 2015). The participation of purinergic signaling is also addressed in retinal diseases (reviewed by Ventura et al., 2019). There is also work related to glaucoma using chicks as a study model (Lauber, 1987; Wahl et al., 2016).

### 1.3 The cAMP pathway

cAMP (Adenosine 3′,5′-Cyclic Monophosphate) is an intracellular second messenger that plays key roles in relaying first messenger information (reviewed by Zaccolo et al., 2021). Hormones, neurotransmitters and other signaling molecules use cAMP to regulate various biological processes, including cellular metabolism, ion channel activation, gene expression, cell growth, differentiation, and apoptosis reviewed by Zhang H. et al., 2020). cAMP is produced from ATP through different adenylyl cyclase (AC) isoforms which are activated by G protein-coupled receptors (GPCRs) (reviewed by Breckler et al., 2011). cAMP has four classes of effectors: Protein Kinase A (PKA), Exchange Protein Directly Activated by cAMP (EPAC), Cyclic Nucleotide Controlled Channels (CNG), and Popeye Domino-Containing Protein (POPDC) (Jakobsen et al., 2019).

**FIGURE 2:**
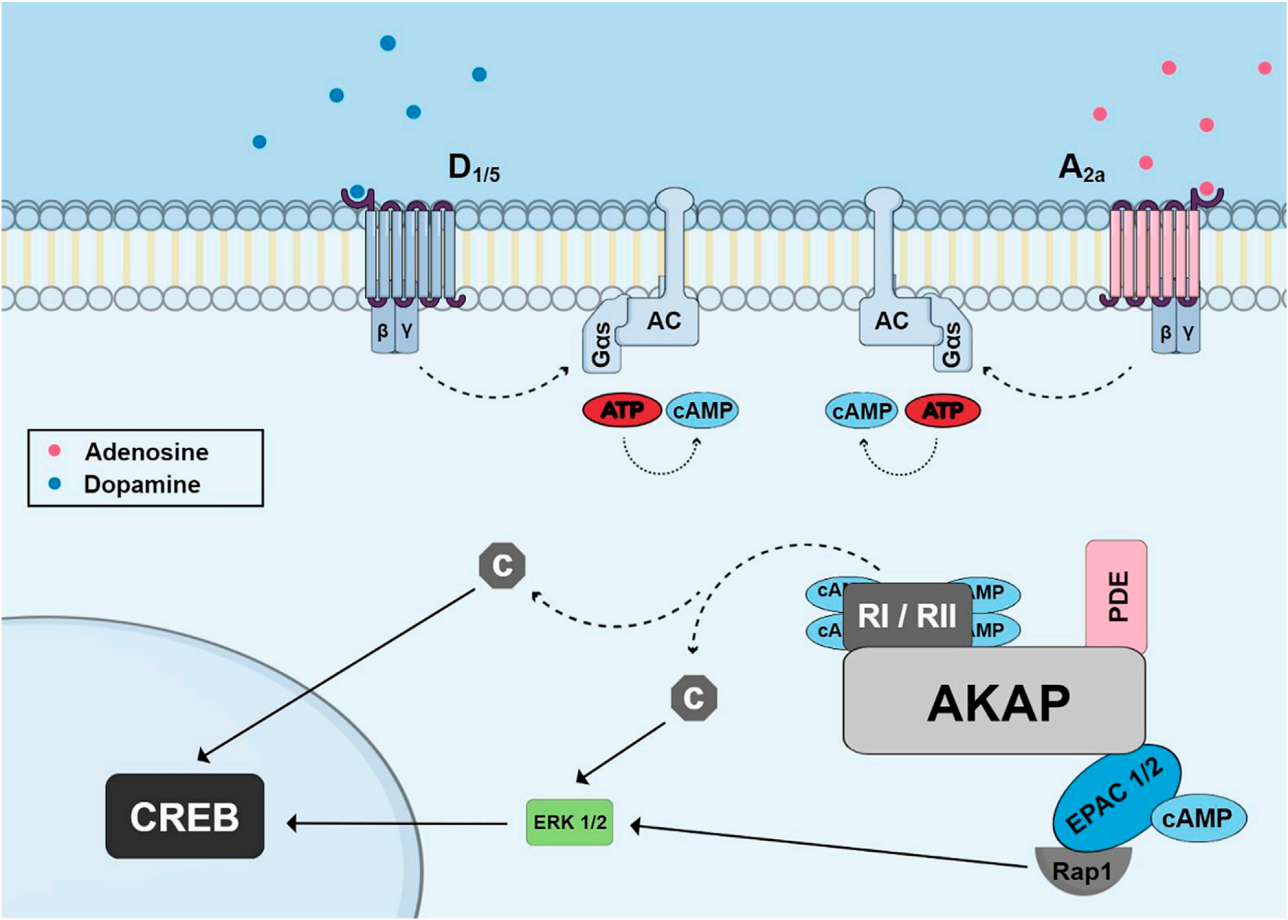
Schematic of cyclic AMP pathway to phosphorylate CREB showing accumulation of cyclic AMP induced by G-protein coupled receptors (dopamine and adenosine receptors) and highlighting the A-kinase Anchoring Protein (AKAP) and its binding sites for PKA regulatory subunits, phosphodiesterase and EPAC proteins. Dissociated catalytic PKA subunits or ERK (activated through EPAC and Rap1) can migrate to nucleus and phosphorylate CREB.

Three main proteins are known to aid in the compartmentalization of cAMP signaling in cells: ACs, Cyclic Nucleotide Phosphodiesterases (PDEs) and Kinase-A Anchoring Proteins (AKAPs) (reviewed by Robichaux and Cheng, 2018). Recent work shows the existence of nanodomains of cAMP in the vicinities of GPCRs leading to independent switches of on/off activity that can eventually fuse to promote different signaling functions (Anton et al., 2022). Nine membrane-bound AC enzymes are expressed in the brain and are regulated by Gs and Gi subunits of the G protein. However, there are other modulators of AC activity, which include calcium/calmodulin, protein kinase C (PKC) and PKA. Phosphodiesterases (PDEs) play an important role in the regulation of the local concentration of cAMP, hydrolyzing it into adenosine 5′-Monophosphate (5′AMP) and cyclic guanosine-3′-5 monophosphate (cGMP) into guanosine 5′-Monophosphate (5′GMP), and this allows the return to basal concentrations of the second messenger after AC stimulation (reviewed by Gerbaud et al., 2016). More than 50 isoforms of PDEs belonging to 11 families (PDE 1-11) have been identified with different enzymatic and regulatory characteristics (reviewed by Calejo and Taskén, 2015). More than 50 AKAP proteins have been characterized and demonstrate the ability to bind PKA regulatory subunits (Autenrieth et al., 2016; Byrne et al., 2022). AKAPs not only present binding sites for PKA, but also to phosphatases, PDEs and other protein kinases (Nijholt et al., 2008) (Figure 2). Of the 50 AKAP proteins, one of the most important is the muscle Anchoring protein A-kinase (mAKAP), also known as AKAP 6 which is a support protein located in the nuclear envelope of neurons and myocytes. mAKAP serves as the coordinator of two cAMP effector pathways to regulate cellular processes and is the only AKAP reported to associate PKA and the exchange protein activated by cAMP type I (EPAC1) (Nijholt et al., 2008). mAKAP displays binding sites for PKA, EPAC1, AC (Type II and V) and Phosphodiesterase 4D (Wang et al., 2015).

PKA, the main effector protein of cAMP, is a serine/threonine kinase (Jakobsen et al., 2019). It was the second protein kinase to be discovered after phosphorylase kinase, a substrate of PKA. PKA is composed of two separate subunits, two catalytic (C) subunits that phosphorylate substrates and two regulatory (R) subunits that bind cAMP (Robichaux and Cheng, 2018). PKA was classically considered the only effector of cAMP, but other targets have been identified, such as EPAC (Nijholt et al., 2008). EPAC proteins function independently of PKA, acting as guanine nucleotide exchange factors (GEFs) specific for Ras GTPase Rap1 and Rap2 family members (Breckler et al., 2011). Two isoforms of EPAC have been identified, EPAC1 and EPAC2. EPAC1 is encoded by the Guanine Nucleotide Exchange Factor Rap3 (RAPGEF3) gene located on chromosome 12 in the human genome. On the other hand, EPAC2 is encoded by the Guanine Nucleotide Exchange Factor Rap 4 (RAPGEF4) located on chromosome 2 in the human genome (Breckler et al., 2011; reviewed by Gao et al., 2022). PKA and EPAC may have synergistic effects, such as in neurite extension, in the regulation of neurotensin secretion, and in the regulation of phosphodiesterases (Cheng et al., 2008). However, PKA and EPAC may have antagonistic effects, including regulation of AKT phosphorylation, acetylcholine regulation, and plasticity (Whitaker and Cooper, 2010).

### 1.4 The cAMP pathway in the retina

The presence of neurotransmitter systems related to cAMP pathways in the developing chick retina is discussed in section 2.1. As stated in the previous section, AKAPs (A-kinase Anchoring Proteins) belong to a family of proteins that play a key role in the intracellular targeting and compartmentalization of cAMP signaling pathways (Nijholt et al., 2008). One study in the retina provides evidence for a neuronal perinuclear cAMP compartment organized by the scaffold protein mAKAPα and shows that this protein is necessary and sufficient for the induction of neurite outgrowth *in vitro* and for the survival of retinal ganglion cells *in vivo* following optic nerve injury (Boczek et al., 2019). PKA is also present in the retina and evidence indicates that it associates with AKAP in the retina (Benz et al., 2020). The Cα subunit is distributed throughout the cell body of all cells in the retina. Cβ subunits are highly enriched in photoreceptors, interneurons, and ganglion cells. The regulatory subunits, RIIα and RIIβ are-located in photoreceptors and interneurons while RIα and Riβ subunits are present in all retinal cells (Roa et al., 2021).

An interesting study also revealed the presence of calmodulin-dependent PDE1 subtypes A, B and C during chick retina development (Deplano et al., 2008). PDE1A is highly expressed at the early stages and decreased as development proceeded. PDE1B expression remained relatively low and constant over time. PDE1C showed a prominent increase (13-fold) between embryonic day (E) 7 and E13, followed by a moderate increase between E13 and postnatal day (P) 1. This differential profile of PDE subtypes suggest distinct functions of these enzymes during chick retina development.

In brain tissue and spinal cord, Epac1 is expressed only during embryonic and neonatal development, whereas EPAC2 is highly expressed in adulthood. In the retina, Epac1 is expressed in the synaptic layers, outer plexiform layer (OPL) and inner plexiform layer (IPL). It is also expressed in the inner nuclear layer (INL) and ganglion cell layer (GCL). EPAC2 is also found in the cell bodies of INL and GCL, as well as in the cell bodies of the outer nuclear layer (ONL) and OPL (Whitaker and Cooper, 2010; Rasmussen et al., 2022).

### 1.5 The AKT pathway

AKT is a serine-threonine kinase classically activated by the PI3K family of enzymes. Receptor tyrosine kinases and GPCRs lead to the recruitment and activation of different classes of PI3K. PI3K class I phosphorylates phosphatidylinositol-4,5-bisphosphate (PI4,5P₂) producing phosphatidylinositol 3,4,5-triphosphate (PIP3) (reviewed by Vanhaesebroeck et al., 2010). This generated substrate can recruit cytoplasmic proteins to the plasma membrane by interacting with the homologous domain of pleckstrin (PH). AKT is a protein that contains a PH domain and, in this way, is recruited to the plasma membrane where it is phosphorylated at its residues Thr 308 and Ser 473 by PDK1 and mTORC2, respectively, and is fully active when phosphorylated at these two residues (reviewed by Fruman et al., 2017). AKT exerts functions in different signaling pathways with many cellular targets. Once activated, it activates several substrates which are involved with cell survival, metabolism, growth, proliferation and migration (Manning and Toker, 2017). The AKT activation process can be regulated by the phosphatase PTEN which dephosphorylates PIP3 to PI4,5P₂.

AKT promotes cell survival in a number of ways, one of which is phosphorylating and inhibiting the BAD protein, a member of the Bcl2 family that exerts pro-apoptotic functions (Datta et al., 2002; reviewed by Maiese et al., 2012). AKT also phosphorylates FOXO, another target phosphorylated by AKT, leading to inhibition of apoptosis. GSK3 can also phosphorylate the transcription factor CREB at the serine-129 residue, suppressing its transcriptional activity (Grimes and Jope, 2001; reviewed by Souder and Anderson, 2019). Inhibition of GSK3 activity by phosphorylation by AKT is then a pathway that can activate CREB. AKT also plays an important role in the activation of mTORC1, an important protein complex that regulates protein synthesis and cell growth (Scott et al., 1998; reviewed by Szwed et al., 2021). The AKT substrate TSC2 (tuberous sclerosis complex 2) downregulates mTORC1 and is inhibited when phosphorylated by AKT, i.e., AKT indirectly activates mTORC1 when it phosphorylates and inhibits TSC2 (Manning et al., 2002; reviewed by Manning and Toker, 2017). These AKT targets also play a role in cell proliferation through the translation and synthesis of proteins involved in the cell cycle (Skeen et al., 2006; reviewed by Wang, 2021).

### 1.6 AKT in the retina

AKT is phosphorylated and can modulate protein synthesis during the cell cycle of retinal progenitor cells in chick embryo retinal cultures (Ornelas et al., 2013). The cyclin D1 protein participates in the cell cycle during the G1 phase and may have its expression increased by the AKT pathway. AKT is also active during the mitosis phase and inhibition of this pathway promotes cell cycle arrest in the G2/M phase (Ornelas et al., 2013). AKT regulates neuronal survival events in the chick retina. One study shows that increased production of nitric oxide (NO) can promote retinal neuronal death in E6 retinas, while in E8 low production of nitric oxide is able to increase neuronal survival. Both events (death in E6 and survival in E8) are dependent on the activity of soluble guanylyl cyclase, PKG and AKT but with different effects on CREB phosphorylation which is inhibited in E6 and stimulated in E8 retinas (Socodato et al., 2014). The PI3K/AKT pathway was shown to be essential for neuronal survival promoted by NO in chick retina neuronal cultures (Mejía-García and Paes-de-Carvalho, 2007). Furthermore, in E8 this signaling pathway promotes the accumulation of AKT in the nucleus promoting regulation of the CREB protein. The CREB signaling pathway is critical in this process because it controls the transcription of survival factors (Socodato et al., 2014). Another study also showed the activation of PI3K and consequent translocation of AKT to the nucleus in chick retina cells in culture stimulated by exogenous NO or stimulation with the NO synthase substrate L-arginine (Mejía-García et al., 2013). This effect of NO is dependent on the sGC/cGMP/PKG pathway which can also be stimulated by the activation of NMDA receptors. Furthermore, NO blocks apoptosis of these cells induced by the toxic stimulus with H₂O₂ (Mejía-García et al., 2013).

In the zebrafish retina, Muller glia cells can regenerate by stimulating the signaling of growth factors that act in an autocrine/paracrine manner. The PI3K signaling pathway participates in this event and must be activated so that Muller glia cells can reprogram, proliferate, and repair the retina in response to a stimulus for injury (Wan et al., 2014). There are also works in the literature showing that PTEN, a phosphatase that controls the activation of the PI3K/AKT pathway, can regulate events in the retina. For example, in PTEN knockout models, the dendrites of amacrine cells are disorganized and AKT phosphorylation is increased in the inner plexiform layer (Sakagami et al., 2012). Furthermore, AKT phosphorylation is important for dendritic development in amacrine cells. The mutation in PTEN also affects the development and differentiation of photoreceptor cells. Studies show that PTEN controls important functions in the retinal circuit (Sakagami et al., 2012; Tachibana et al., 2016).

### 1.7 CREB as a hub of signaling pathways

CREB (cyclic nucleotide-responsive element binding protein) is an important transcription factor involved in many functions during CNS development such as survival and differentiation of neurons (reviewed by Belgacem and Borodinsky, 2017), as well as in cognition and memory (Morè et al., 2022).

CREB is localized in cell nuclei and is stimulated by phosphorylation of residue ser 133 by several intracellular signaling pathways including the classical cAMP/PKA but also by ERK/RSK, AKT/GSK3b and Calcium/CAMKIV (West et al., 2001; Bengtson and Bading, 2012; reviewed by Ahmed et al., 2022). In this way, it can be considered a signaling hub, converging distinct regulatory elements. Among the more studied genes regulated by CREB are the genes for antiapoptotic proteins and BDNF (Esvald et al., 2020).

### 1.8 CREB in the retina

**FIGURE 3:**
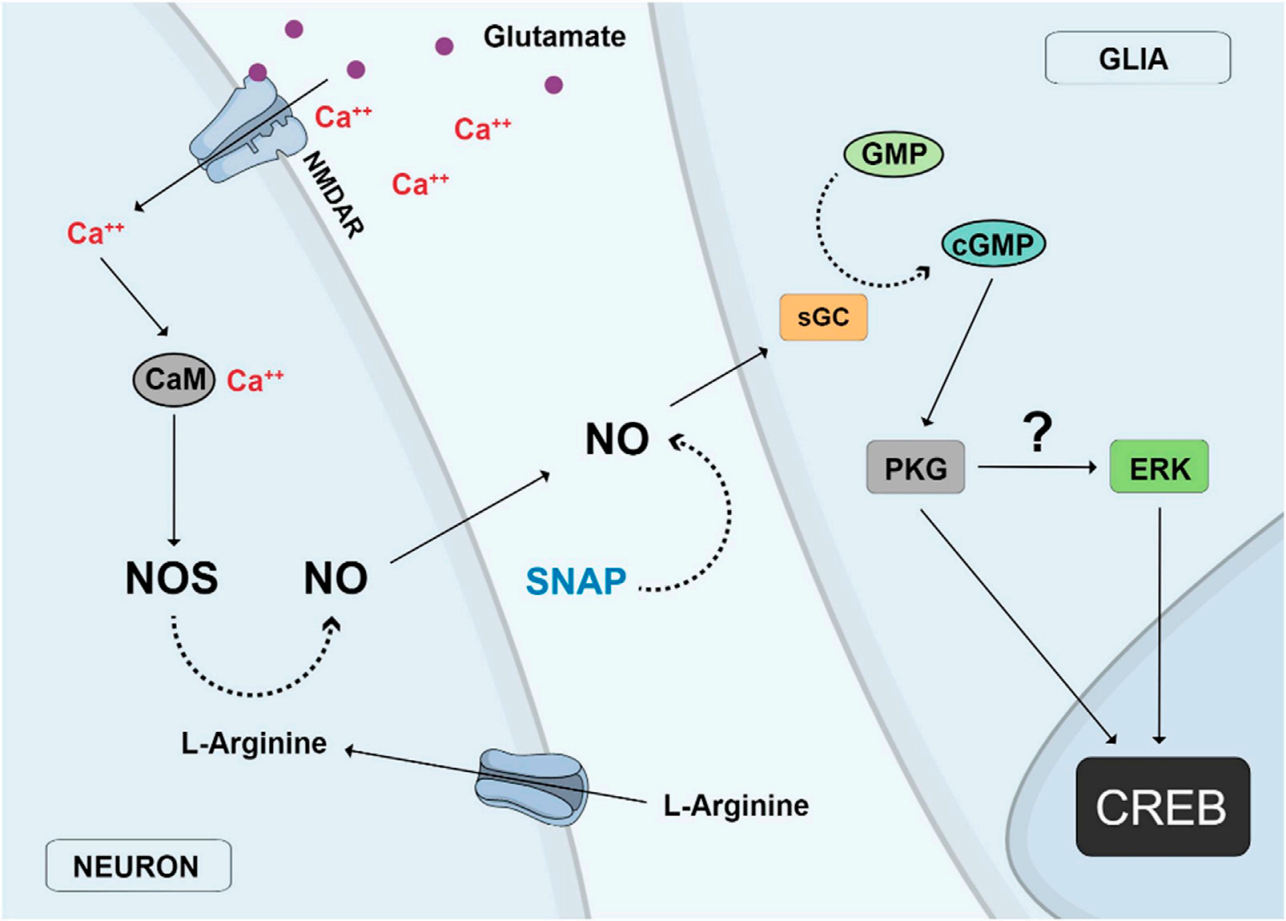
Nitric oxide (NO) neuronal-glial cycle. Activation of glutamate NMDA receptors (NMDAR) promotes calcium influx and stimulation of the calcium/calmodulin enzyme nitric oxide synthase (NOS) and NO production in neurons. NO diffuses out from neurons to glial cells where it stimulates soluble guanylyl cyclase (sGC) to produce cyclic GMP which activates protein kinase G (PKG), which then stimulates Erk and finally CREB in glial cell nuclei. The same effect is observed with the addition of the NO donor SNAP or the uptake of L-arginine, the substrate of NOS.

Activation of glutamate NMDA receptors can activate ERK and CREB in chick retina cells through the accumulation of NO and stimulation of the classical guanylyl cyclase/cGMP/PKG pathway (Socodato et al., 2009). In this case, CREB activation by glutamate was found to occur in glial cell nuclei in mixed retinal cultures containing neurons and glial cells, whereas a direct CREB stimulation can be accomplished by the NO donor SNAP, implicating that NO can be produced by glutamate stimulation of neurons and released to activate CREB in glial cells (Figure 3). Ascorbate can also stimulate CREB in retinal cultures but this effect is mediated by glutamate and stimulation of NMDA receptors (Domith et al., 2018b). One study showed that nucleotides regulate CREB activity and proliferation of progenitor cells in chick retina cultures through the P2Y13 subclass of P2 purinergic receptors (Jacques et al., 2017). Moreover, as described before, CREB phosphorylation or dephosphorylation by different pathways is fundamental for neuronal survival choices during chick retina development (Socodato et al., 2011, 2014).

## 2 Neurotransmitters and receptors in the retina

Most if not all neurotransmitter or neuromodulator systems present in other CNS regions are also present in the chicken retina, including some already described to stimulate GPCRs coupled to adenylyl cyclase such as dopamine (de Mello, 1978), adenosine (Paes-de-Carvalho and de Mello, 1982), PACAP (Fleming et al., 2013), as well as neurotransmitters linked to calcium and AKT metabolism as glutamate (Romano et al., 1998). Several neurotransmitter receptors appear very early during development in a profile consistent with the idea that some of these molecules and related neuromodulators can also function as morphogenic factors and developmental signals to regulate embryological phenomena such as neurite outgrowth and synapse formation (Lauder, 1987, 1988). Here we will discuss some of the neurotransmitter and neuromodulator systems more studied in the developing chick retina with the emphasis in the cAMP and AKT signaling pathways triggered by the activation of their specific receptors.

### 2.1 Neurotransmitter systems coupled to adenylyl cyclase in the retina

#### 2.1.1 Dopamine

Dopamine is an important neurotransmitter involved in several processes in the CNS, such as modulation of motor control, reward mechanisms and endocrine functions, as well as neurological and psychiatric disorders (reviewed by in Klein et al., 2018).

Dopamine is a major catecholamine present in retinas of several species (reviewed by Korshunov et al., 2020). In the avian retina, dopaminergic cells are found primarily in the inner nuclear layer (INL), which contains the cell bodies of amacrine neurons, whose processes extend exclusively to the inner plexiform layer (IPL) (Araki et al., 1983).

Dopamine receptors belong to the G protein-coupled receptor superfamily and are classified into two subfamilies based on their biochemical characteristics: D1-type receptors (comprising D1 and D5 receptors) and D2-type receptors (comprising D2, D3, and D4 receptors) (Mishra et al., 2018). D1-type receptors promote activation of adenylyl cyclase (AC) *via* Gs, consequently increasing the intracellular content of cAMP in retinas from several species (Figure 2). On the other hand, D2-type receptors inhibit AC activity by activating Gi or Go (Ventura and de Mello, 1990; Reis et al., 2007).

Some studies have shown dramatic variations of the cAMP content during chick embryo retina development. The basal cAMP level is low from the embryonic day 6 (E6) up to E15 but increases 3 times between E15 and E17. On the other hand, stimulation with dopamine in E7 promotes an increase in cAMP concentration of 5 times above basal level. Moreover, a 20-fold increase was observed between E8 and E16 (de Mello, 1978). Then, stimulation of dopamine receptors promotes activation of AC since E7, with a maximum observed in E14 (Lankford et al., 1988). Similarly, forskolin, a potent and direct activator of adenylyl cyclase, promotes an increase of cAMP during the period from E8 to E13, stabilizes up to E18 and decreases from E19 onwards (Paes-de- Carvalho and de Mello, 1982).

**FIGURE 4:**
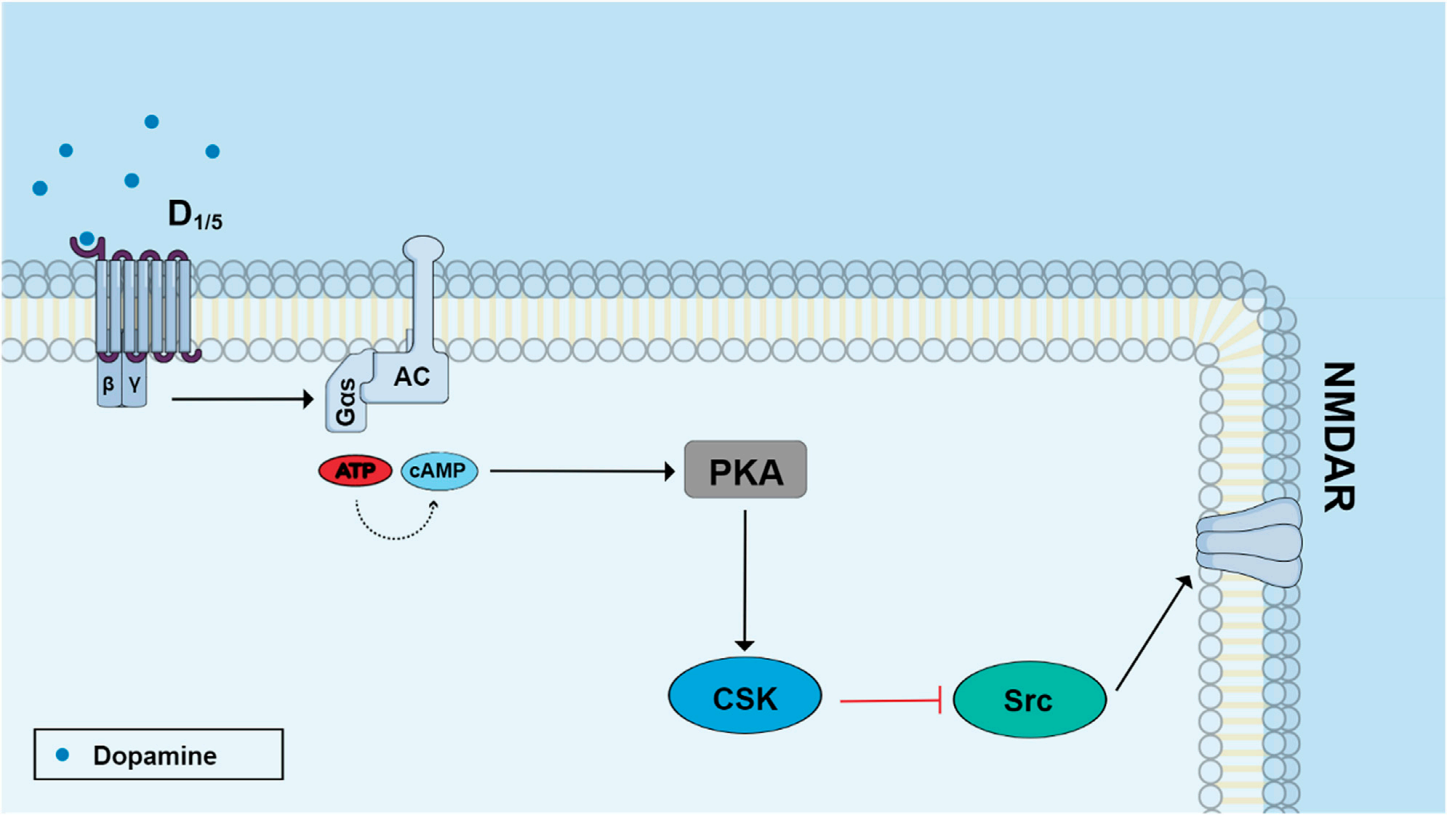
Dopamine regulates NMDA receptor function through the PKA/CSK/Src pathway. Stimulation of D1-like receptors with dopamine activates adenylyl cyclase and increases cyclic AMP production. Activated protein kinase A (PKA) induces the phosphorylation of C-terminal Src kinase (ser 364 Csk) which phosphorylates Tyr 527 Src leading to enzyme inhibition. Src phosphorylates the subunit 2B of NMDA receptor in the residue Tyr 1472 and then Src inhibition leads to diminished receptor activity.

Dopamine was shown to inhibit growth cone motility and neurite outgrowth of retinal neurons in culture *via* D1 receptors and cAMP (Lankford et al., 1988), corroborating the idea that neurotransmitters are also morphogenic signaling molecules. Dopamine is also involved in regulating glutamate NMDA receptors through a PKA/CSK/Src pathway (Figure 4). Dopamine activates D1 receptors and a cAMP/PKA cascade which leads to a PKA-dependent phosphorylation of a tyrosine kinase named CSK (C-terminal Src Kinase) (Socodato et al., 2017). This kinase catalyzes the phosphorylation of residue tyr527 of Src kinase and promotes enzyme inhibition (Masato, 2012). Interestingly, this effect promotes inhibition of Src kinase-dependent posphorylation of NMDA subunit 2B and decreases receptor activity in embryonic retinal cells in culture (Socodato et al., 2017).

The presence of these systems in early stages of development suggests that they could have important functions on neuronal development. Indeed, dopamine regulates neurite growth in retinal cultures but the effects of these changes in more developed neurons are not known (Lankford et al., 1988).

**FIGURE 5:**
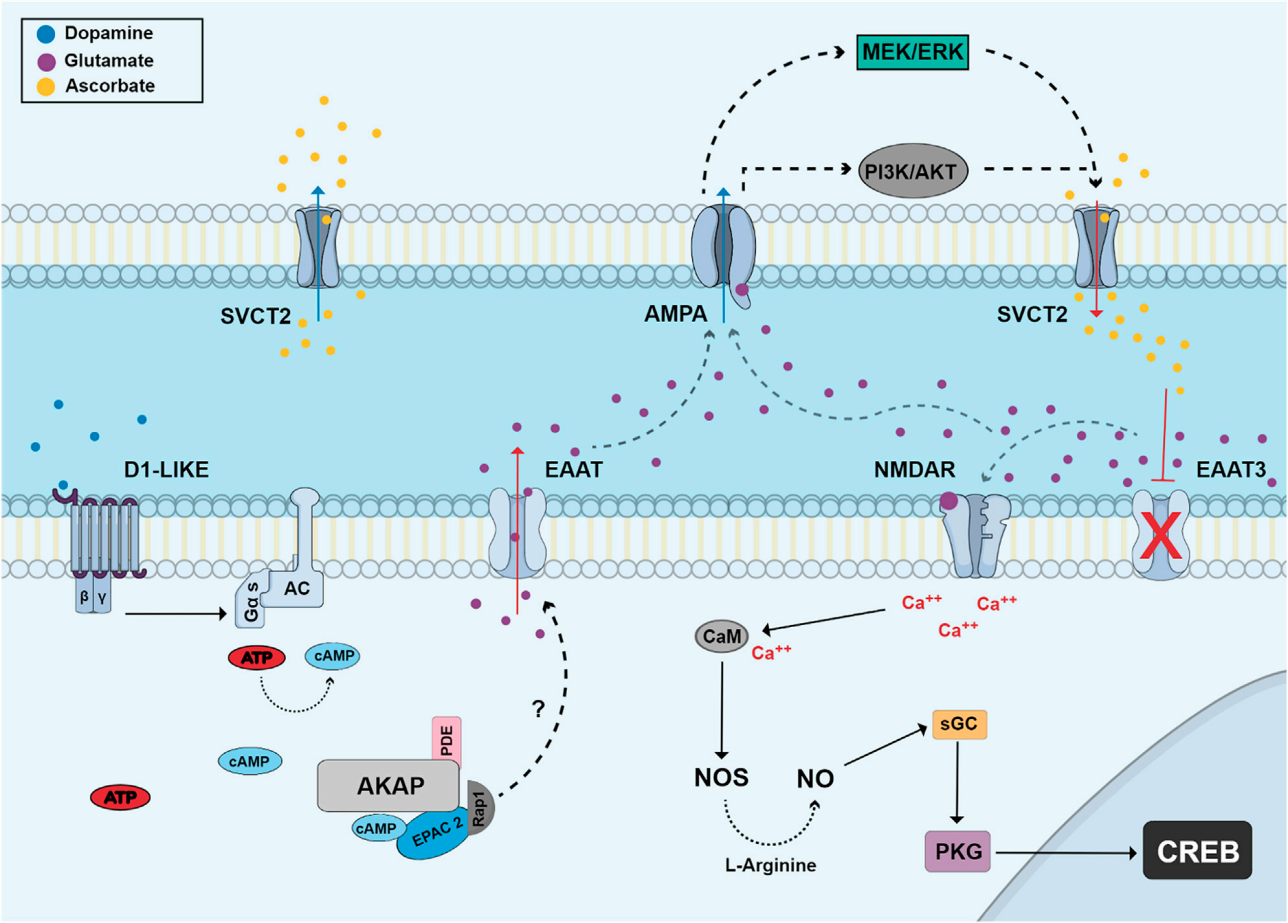
Dopamine regulates the release of vitamin C *via* EPAC and glutamate receptors. Dopamine stimulates D1-like receptors and activates adenylyl cyclase and cAMP production. cAMP stimulates EPAC2 and Rap1 which stimulates the release of glutamate by an unknown mechanism. Glutamate can then stimulate AMPA receptors in a second cell activating MEK/ERK and PI3K/AKT pathways and promoting ascorbate efflux through the sodium/vitamin C transporter 2 (SVCT2). Extracellular ascorbate promotes an increase of extracellular glutamate by inhibiting EAAT3 activity and then glutamate can stimulate NMDA receptors (NMDAR). This activation leads to calcium influx and increased NO production by calcium/calmodulin-dependent NOS, which then can stimulate the canonical PKG-dependent pathway and CREB phosphorylation.

Another interesting function of dopamine in retina is the modulation of vitamin C release. Dopamine *via* D1 receptors and the cAMP/EPAC pathway is able to promote the release of ascorbate in retinal cultures (da Encarnação et al., 2018). This effect of dopamine was later shown to be mediated by the release of glutamate and activation of AMPA/kainate receptors (Portugal et al., 2019) (Figure 5).

#### 2.1.2 Adenosine

Adenosine is a nucleoside that plays an important role as a neuromodulator or neurotransmitter in the CNS (reviewed by Schulte and Fredholm, 2003). It is metabolized by deamination or phosphorylation by the enzyme adenosine deaminase or adenosine kinase, respectively, and activates receptors coupled to AC in the retina during different stages of development. Four receptor subtypes, all coupled to heterotrimeric G proteins, are presently known, with subtypes A1 and A3 being classically coupled to Gi, inhibiting AC activity, while subtypes A2a and A2b are coupled to Gs protein, stimulating AC.

##### 2.1.2.1 Regulation of cyclic AMP accumulation by adenosine receptors

**FIGURE 6:**
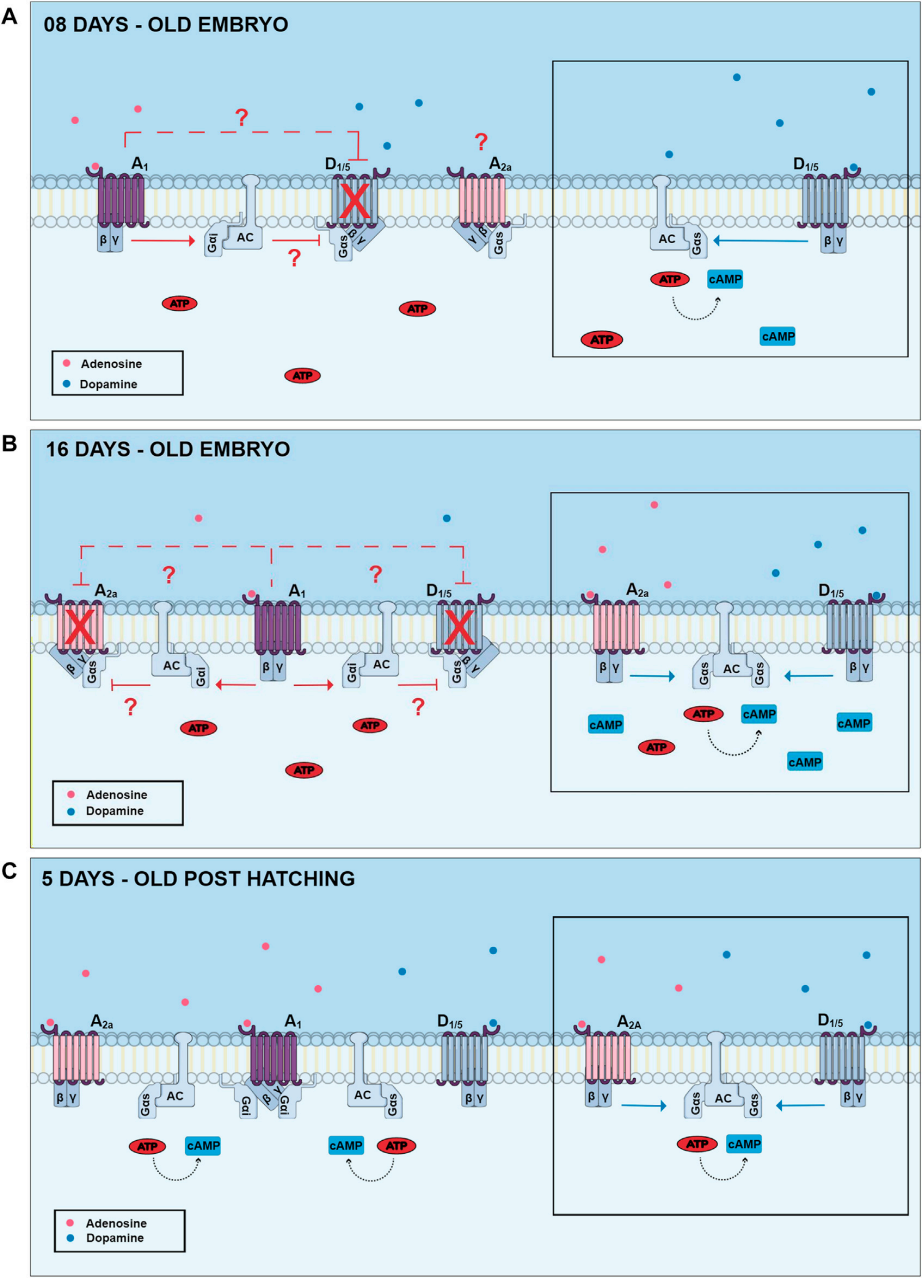
Changes of dopamine and adenosine-dependent cAMP levels in three different developmental stages of the chick retina. In E8 retinas, dopamine promotes a high increase of cAMP *via* stimulation of D1-like receptors (lateral picture), and this effect can be inhibited by activation of adenosine A1 receptors. Adenosine A2a receptors are present in this period but are not coupled to cAMP production. In E16 retinas, both D1 and A2a receptors are present and promote cAMP accumulation. However, cAMP increase is not additive due to the presence of A1 inhibitory receptors. In post-hatching retinas, dopamine and adenosine promote a lower increase of cAMP accumulation and A1 receptor-mediated decrease of dopamine-dependent cAMP accumulation is no longer observed. Gαi and Gαs are respectively inhibitory and stimulatory Gα protein subunits; AC, adenylyl cyclase.

As mentioned before, adenosine receptors have been shown to regulate AC activity and cAMP levels. In chick embryo retinas from E8 to E13, no increase in cAMP levels was observed when exposed to adenosine. Interestingly, embryos from E14 to E17 days showed a gradual adenosine-dependent increase in cAMP levels, reaching the maximum level in E17. In addition, in experiments carried out in post-hatch animals, lower levels of stimulation with adenosine were observed (Paes-de-Carvalho and de Mello, 1982). These results suggest that the increase of cAMP levels observed after stimulation of chick retinas may vary according to developmental stages (Figure 6) (reviewed by Paes-de-Carvalho, 2002).

In addition to adenosine receptors, dopamine receptors are also capable of promoting an increase in intracellular cAMP levels. One study demonstrated that in E12 chick embryo retinas, activation of D1-type dopamine receptors promotes an increase in cAMP levels, but that this effect was blocked by the use of adenosine A1 receptor agonists (Figure 6) (Paes-de-Carvalho and de Mello, 1985). Interestingly, this blocking effect upon dopamine-dependent cAMP accumulation is no longer observed in post-hatching chick retinas, but an inhibition of a cAMP increase induced by forskolin is still present (Paes-de-Carvalho, 1990). One explanation for this effect could be a developmental decrease of D1/A1 heteromers, but this possibility remains to be investigated.

##### 2.1.2.2 A1 adenosine receptor

Classically, adenosine A1 receptors are associated with the Gi protein, which inhibits AC activity, leading to a decrease in intracellular cAMP levels. However, it is already known that the adenosine A1 receptor also has the ability to modulate protein kinase C (PKC) activation (Di-Capua et al., 2003). A study using mice showed that when exposed to chronic intermittent hypoxia, the animal underwent morphological changes and a process of apoptosis in hippocampal neurons, generating a cognitive deficit. Moreover, activation of adenosine A1 receptors was able to play a neuroprotective role, in addition to modulating the Gα(i)-cAMP-PKC pathway, promoting the formation of LTP and increased synaptic plasticity (Zhang Y. et al., 2020).

In the chicken retina, adenosine A1 receptors appear throughout development at levels that vary according to embryonic age. Binding studies using [^3^H] Cyclohexyl adenosine (CHA), an A1 receptor agonist, showed an expression of these receptors in the first stages of development, but at very low levels. However, throughout development, the levels of these receptors gradually increase, reaching a peak in E16. In post-hatching animals, A1 receptor levels decrease. Autoradiographic data showed the presence of these receptors in the inner and outer plexiform layers since E12 and high labeling was observed in E18 as well in post-hatched retinas (Paes-de-Carvalho et al., 1992).

It has already been described in the literature that cell aggregation and cAMP/PKA pathways regulate A1 receptor expression in cultured retinal cells (Pereira et al., 2010). Moreover, long-term activation of adenosine A2a receptors, which promote cAMP accumulation, also has the ability to regulate A1 receptor expression in retinal cells, suggesting that regulation of extracellular adenosine levels is a key factor capable of controlling adenosine receptor expression (Pereira et al., 2010). Interestingly, long-term exposure of retinal cell in culture to adenosine promotes a decrease of cAMP accumulation stimulated by adenosine (de Mello et al., 1982).

One recent study raised the possibility that exposure to caffeine, a non-selective antagonist of adenosine A1 and A2a receptors, during the intermediate stages of development, leads retinal cells to a more ischemic-resistant state. The precise mechanism by which caffeine protects the retina still needs further study. The data presented indicate that tissue excitability modulated by the GABAergic and glutamatergic systems appears to play an important role. This protective mechanism appears to be triggered by adenosine A1 receptor, CREB phosphorylation and BDNF production (Pereira-Figueiredo et al., 2020).

##### 2.1.2.3 A2a adenosine receptor

A2a receptors are present in the retina of several animals, including rats, mice and chickens (Paes-de-Carvalho et al., 2003; Li et al., 2013). Several studies show a preeminent role of A2a receptors in the outer retina (McIntosh and Blazynski, 1994; Li et al., 2013; Huang et al., 2014; Cao et al., 2021). However, A2AR-immunoreactivity was shown to be expressed as puncta in the ganglion cell layer, inner plexiform layer, inner nuclear layer, and outer retina of the zebrafish (Grillo et al., 2019).

**FIGURE 7:**
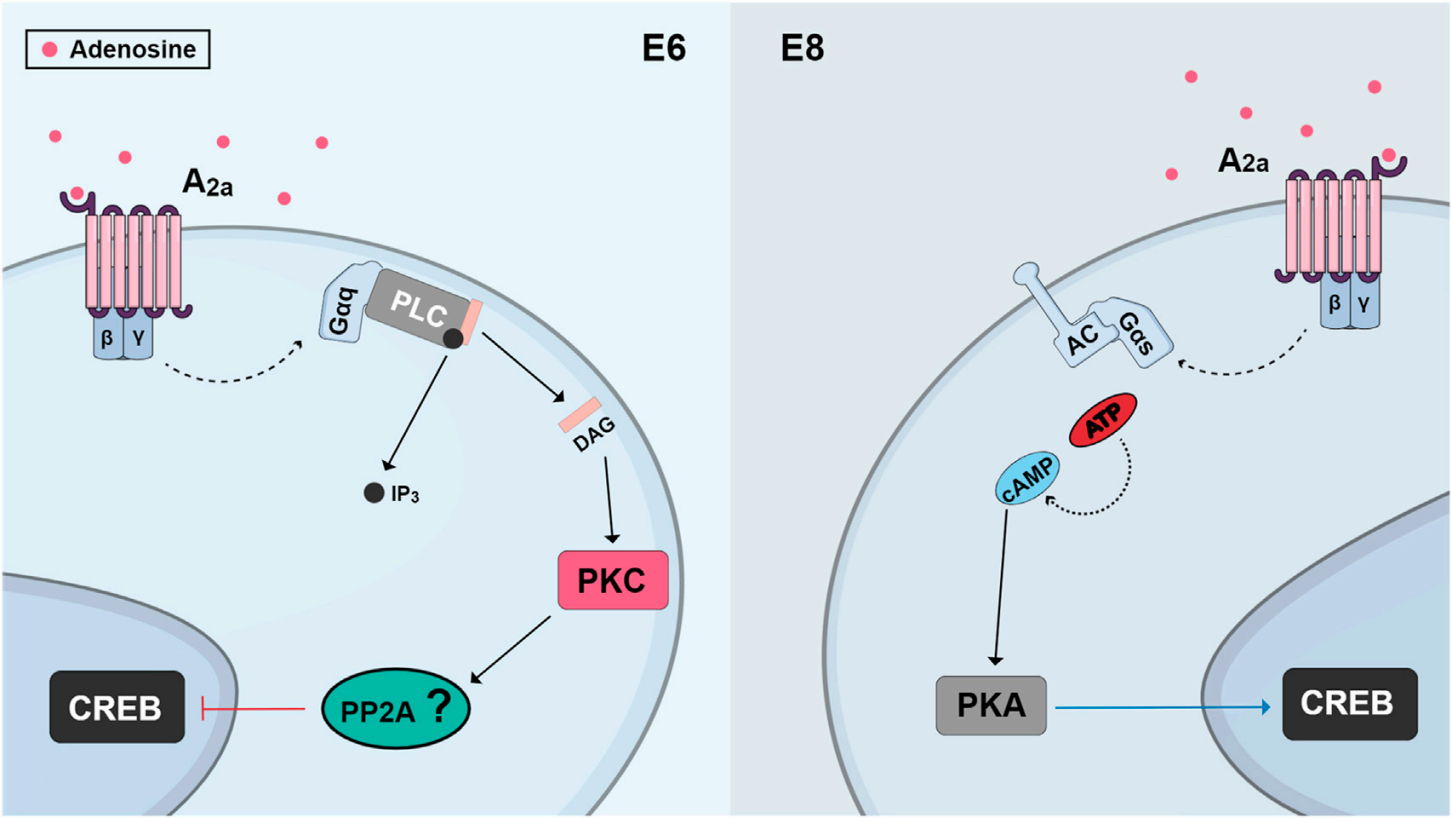
Schematic view of changes observed in CREB regulation by A2a receptors in different ages of cultures from developing chick retina. In cultures obtained from E6 retinas, activation of A2a receptors promotes phospholipase C/PKC stimulation and CREB dephosphorylation, probably through the activation of a phosphatase, leading to cell death. On the other hand, in cultures from E8 embryos, activation of A2a receptors promotes CREB phosphorylation, through a cAMP/PKA pathway, and cell survival.

In purified cultures of retinal neurons, adenosine is neuroprotective against glutamate excitotoxicity or cell death induced by feeding cells with fresh medium. This effect is mediated by the activation of A2a receptors and accumulation of cyclic AMP, but it is only observed when these receptors are activated by long-term treatments of at least 24 h (Paes-de-Carvalho et al., 2003). Interestingly, as described in the previous section, these long-term treatments can promote increases in A1 receptor expression (Pereira et al., 2010), raising the possibility that the neuroprotective effect is in fact mediated by A1 receptors. Indeed, in chick retina cultures obtained from E8 embryos, activation of adenosine A2a receptors and the cAMP/PKA pathway is neuroprotective and promotes CREB phosphorylation (Socodato et al., 2011). However, activation of these receptors causes massive cell death in cultures obtained from E6 retinas, an effect mediated by increased activation of PKC and dephosphorylation of CREB (Figure 7). Activation of these receptors also can exhibit cytotoxic effects in the embryonic retina *in vivo*, where it will induce CREB dephosphorylation. These results suggest that the regulation of CREB activity and retinal neuronal survival by adenosine depends on the period of development and activation of different signaling pathways (Socodato et al., 2011).

##### 2.1.2.4 Adenosine A3 and A2b receptors

As previously reported, the adenosine A3 receptor is coupled to a Gi protein, inhibiting the activity of AC and consequently leading to a decrease in intracellular cAMP levels. Some studies show the presence of this receptor in retinal ganglion cells (RGCs). Findings related to this receptor suggest its contribution to the reversal of the toxic effect observed after stimulation of the P2X7 type of ATP receptor in RGCs (Zhang et al., 2006). When activated, the A3 receptor has the ability to modulate NMDA-dependent calcium influx into ganglion cells (Zhang et al., 2010) and also promotes neurite outgrowth *in vitro* and *in vivo* during the regeneration of mouse retinal ganglion cells. This latter effect is caused by the activation of an Akt-dependent signaling pathway (Nakashima et al., 2018).

The expression of the A3 receptor in the developing chicken retina model is still poorly characterized. A study performed in chicken retina culture showed that the adenosinergic system regulates the bioavailability of vitamin C in primary neurons and that the activation of A3R can increase the release of ascorbate in a Sodium-Vitamin C transporter 2 (SVCT2)-dependent manner. This decrease of ascorbate content leads to an accumulation of reactive oxygen species, showing an essential and specific role of the adenosinergic system in the control of vitamin C homeostasis in a way that directly affects neuronal redox balance (Portugal et al., 2021). Although these are promising results, more studies are still needed to show the roles played by the A3-type adenosine receptor in the retina.

The adenosine A2b receptor is a G protein-coupled receptor and is activated by high concentrations of extracellular adenosine mediated by inflammation or hypoxia, for example (reviewed by Sepúlveda et al., 2016). This receptor, in addition to associating with Gs, also can associate with Gq, triggering an activation of phospholipase C and the mobilization of calcium in different animal models (reviewed by Linden, 1991). Some studies show that the A2b receptor is overexpressed in several types of tumors and in events such as angiogenesis and metastasis, which could be a suggestion that the receptor could promote the progression of these tumors (Sepúlveda et al., 2016). Regarding the A2b receptor, little is known about its presence in the chicken retina, and further studies are needed for a better characterization of this receptor and its effects in this animal model.

#### 2.1.3 Neuropeptides and cannabinoids

Pituitary Adenylyl Cyclase-Activating Polypeptide (PACAP) is considered as a neuroprotective agent in several CNS tissues including in the retina of several species (Szabadfi et al., 2012). In the avian retina, PACAP activates AC since E8, and this effect desensitizes after E12. As also shown for dopamine-dependent cAMP accumulation (Paes-de-Carvalho and de Mello, 1982), chronic administration of PACAP or the PACAP antagonist (PACAP 6-38) regulates PACAP receptor/cyclase system *in vitro* and *in vivo*. Interestingly, the peptide is also able to regulate the expression of tyrosine hydroxylase positive (TH⁺) cells, a marker of dopamine containing cells (Fleming et al., 2013).

Cannabinoid receptors are present in the retinas of several species (Straiker et al., 1999). In the chicken retina, CB_1_ receptor is highly expressed from embryonic day 5 (E5) until post hatched day 7 (PE7), decreasing its levels throughout development. CB_1_ is densely found in the ganglion cell layer (GCL) and inner plexiform layer (IPL). CB_2_ receptor was also found from E5 until PE7 with a decrease in its contents from E9 afterwards (da Silva Sampaio et al., 2018). CB_1_ is co-localized with TH and was heavily associated to dopamine D_1_ receptor labeling in primary cell cultures. In chick retina cultures, cAMP accumulation was stimulated by the selective D_1_ agonist SKF38393, and this effect is inhibited when cultures were treated with WIN55, 212-2 (WIN) in a CB_1_- dependent manner. The results suggest a correlation between the endocannabinoid and dopaminergic systems (DSs) during avian retina development. Activation of CB_1_ limits cAMP accumulation *via* D_1_ receptor activation and may influence embryological parameters during avian retina differentiation.

## 3 Glutamate and related neuromodulator systems

### 3.1 Glutamate

The main route of glutamate synthesis in neurons is from glutamine. Glia are able to release glutamine to the neuron, which through the enzyme glutaminase converts glutamine into glutamate (reviewed by Newsholme et al., 2003). Another route of glutamate synthesis is through the mitochondrial enzyme glutamate dehydrogenase (GDH), converting α-ketoglutarate to glutamate (Dewan, 1938). Interestingly, some compounds can interfere with glutamate synthesis by regulating GDH activity. Some of them are routinely consumed in beverages around the world, such as chlorogenic acids, found in coffee, and some green tea polyphenols such as epigallocatechin gallate and epicatechin gallate (Li et al., 2006, 2011; Domith et al., 2018a).

Glutamatergic signaling occurs through a wide range of receptors. It acts on ionotropic receptors, which include NMDA (N-methyl-D-Aspartate), AMPA (alpha-amino-3-hydroxy-5-methyl-4-isoxazole-propionate) and kainate receptors (Wyllie and Bowie, 2022), as well as metabotropic receptors (reviewed by Watkins and Evans, 1981; and Ferraguti et al., 2022; Mayer and Westbrook, 1987; De Blasi et al., 2001).

### 3.2 Glutamate in the retina

As in other areas of the CNS, glutamate is also the main excitatory neurotransmitter in the retina (Mayer and Westbrook, 1987; Rauen, 2000; Bui et al., 2009; reviewed by Boccuni and Fairless, 2022). In the chick retina, a complex neurochemical network regulated by glutamate is present in the developing as well as in the mature tissue (reviewed by Paes-de-Carvalho et al., 2008). The presence of glutamate receptors was observed mainly in amacrine and ganglion cells, in addition to the inner and outer plexiform layers (Silveira Dos Santos Bredariol and Emi Hamassaki‐Britto, 2001). Also, NMDA receptors has been observed in Müller cells, the only type of glial cell found in chick retina (Lamas et al., 2005).

Glutamate ionotropic receptors signaling in chick retina is involved in a wide range of cellular effects and many of these effects involve calcium signaling. In cultures of chick retina cells obtained from E8 embryos, glutamate, kainate or AMPA treatment decreases neurite outgrowth (Catsicas et al., 2001). This inhibition was due to activation of AMPA receptors, since pre-incubation of these cultures with CNQX or GYKI 52466, AMPA receptors antagonists, but not with AP5, an NMDA receptor antagonist, did prevent the inhibitory effect. Moreover, Ca^2+^-permeable AMPA receptors were involved since the selective antagonist JSTX-3 was also able to block this effect (Catsicas et al., 2001). Another event involving Ca^2+^ observed in chick retina is the modulation of NMDA receptor by caffeine. Mixed retinal cultures treated with caffeine increased basal NMDA receptor activity. In addition, if these cells were pre-treated with caffeine and then stimulated with NMDA, there was an increase in intracellular Ca^2+^ when compared to NMDA alone (Pereira-Figueiredo et al., 2020).

As caffeine, vitamin C also modulates NMDA receptor activity in the retina. Ascorbate, the reduced form of vitamin C, and dehydroascorbate, the oxidized form, increased NMDA receptor function in the absence of glutamate, an effect observed by measuring the increase in [^3^H]-MK-801 binding in chick retina mixed cultures (Domith et al., 2018b). Glutamate, as expected, increased the binding to a much greater extent as [^3^H]-MK-801 binds to an open state channel conformation. Interestingly, if the cultures were treated concomitantly with glutamate plus ascorbate or dehydroascobate, [^3^H]-MK-801 binding was lower when compared to glutamate treatment alone (Domith et al., 2018b). Moreover, data also show that ascorbate reduces NMDA receptor levels in the cell membrane, suggesting that this vitamin prevents receptor over-activation. In addition, ascorbate started a NMDA receptor and calcium-dependent downstream signaling pathway that culminated in an increase of CREB phosphorylation (Domith et al., 2018b). Overall, these data suggest vitamin C as a NMDA receptor signaling modulator (see below).

Some studies show a relationship between glutamatergic signaling and other neurotransmitters in chick retina (Passos et al., 2019). L-glutamate decreases serotonin uptake, an effect blocked by the AMPA/kainate antagonist CNQX, but not by the NMDA receptor antagonist MK-801. In addition, this inhibitory effect was not observed in the presence of AMPA, suggesting the involvement of kainate receptors (Passos et al., 2019). Some data also support an interaction between NMDA receptors and cannabinoid signaling. Kubrusly and collaborators (2018) described in chick retina that after L-aspartate exposure, there was less GABA^+^ amacrine cells, and that pre-incubation with the cannabinoid receptor agonist WIN 55,212-2 inhibited this effect. These data suggest the existence of some interaction between CB1 and/or CB2 and NMDA receptors in GABA release (Kubrusly et al., 2018).

Interestingly, NMDA receptors also not only modulate NO production but protein synthesis in chick retina cells (Cossenza and Paes-de-Carvalho, 2000; Cossenza et al., 2006, 2014; Gladulich et al., 2020). Stimulation of NMDA receptors activates the Ca^2+^/calmodulin-dependent enzyme eEF2K, a kinase that phosphorylates the elongation factor eEF2 and inhibit its activity, promoting a decrease of protein synthesis rate (Cossenza and Paes-de-Carvalho, 2000; Cossenza et al., 2006, 2014; Gladulich et al., 2020). Experiments utilizing arginine-free solutions demonstrated that inhibition of eEF2K also promotes an increase of NMDA-dependent NO production (Gladulich et al., 2020). This effect would be due to an increase of arginine availability for NO production since this amino acid would be less directed towards protein synthesis. Some data showed that the protein Homer1b/c plays an important role in this protein synthesis regulation, since lack of this protein impairs this signaling pathway (Gladulich et al., 2021).

Metabotropic glutamate receptors (mGluRs) are divided into different subtypes that modulate several signaling pathways through G proteins. Different G protein isoforms have varied effects, so that they can promote activation (G_s_) or inhibition of adenylyl cyclase (G_i_), activation of phospholipase C (G_q_), among others. Group I mGluRs comprise mGluR1 and mGluR5, coupled to the Gq protein, while group II receptors comprise mGluR2 and mGluR3 receptors, coupled to the Gi or Go protein; group III receptors comprise the mGluR4, mGluR6, mGluR7 and mGluR8 receptors, and may also be associated with Gi or Go (De Blasi et al., 2001; reviewed by Gregory et al., 2011; and Reiner and Levitz, 2018).

Previous evidence shows that activation of chick retina mGluR from group III inhibits cAMP formation during the embryonic period until hatching, demonstrating its presence and functionality early in development (Sampaio and Paes-de-Carvalho, 1998). Interestingly, presynaptic mGluR4/7 increases glutamate release in chick retina, which in turn activates postsynaptic ionotropic glutamate receptors, promoting Na + influx and GABA release *via* GAT1 (Guimaraes-Souza and Calaza, 2012). In addition, some evidence suggests that postsynaptic mGluR8 actually decreases GABA release (Guimaraes-Souza and Calaza, 2012). In relation to group I, some data indicates that mGluR5 in amacrine cells of avian retina are involved in Ca+2 release and influx *via* phospholipase C (Sosa et al., 2002). Glutamate transport occurs mainly *via* sodium-dependent excitatory amino acid transporters (EAAT). There are five isoforms of EAATs: isoforms 1 (EAAT1 or GLAST) and 2 (EAAT2 or GLT1) glial, isoform 3 (EAAT3 or EAAC1) found in neurons, isoform 4 (EAAT4) found in Purkinje cells of the cerebellum (reviewed by Shigeri et al., 2004) and isoform 5 (EAAT5) found in retina (Arriza et al., 1997; reviewed by Magi et al., 2019). There are different levels of expression of EAATs isoforms 1, 2, 3 and 4 during human development (Bar-Peled et al., 1997). During the embryonic phase, EAAT2 is the most expressed isoform in different regions of the brain, such as cortex and hippocampus (Bar-Peled et al., 1997). In the frontal cortex, expression of EAAT2 increases significantly, while in the same region EAAT4 decreases from the embryonic stage to the postnatal and adult stages (Bar-Peled et al., 1997).

In general, EAATs promote glutamate uptake, but interestingly, it has been shown that during ischemic events, the reversal of glutamate transporters can occur, causing cell death by activating NMDA receptors (Rossi et al., 2000; Camacho and Massieu, 2006). In chick retina culture, ascorbate promotes a decrease in EAAT3 surface levels in neurons, leading to glutamate accumulation at the extracellular space, but not promoting cell death in acute treatment (Domith et al., 2018b). As mentioned before, this glutamate accumulation activates NMDA receptor signaling pathway. Interestingly, EAAT1 D-aspartate transport increases the Na+/Ca+2 exchanger activity which starts a Ca+2-dependent signaling pathway in Müller glia cells of chick retina (López-Colomé et al., 2012). This Ca+2 influx results in mTOR phosphorylation in a SRC and AKT-dependent manner (López-Colomé et al., 2012).

Overall, it is possible to verify that glutamate is an important neurotransmitter in the chick retina. The data clearly demonstrate its role in cell signaling with emphasis on calcium-dependent pathways.

### 3.3 Vitamin C

Vitamin C can be found in two forms, ascorbate, its reduced form, and dehydroascorbate, its oxidized form. Humans are not able to synthesize vitamin C, having to acquire it through the consumption of fruits and vegetables during the diet. It is estimated that 100 mg/day is sufficient to meet physiological needs. Ascorbate is absorbed in the intestine and distributed to tissues by the specific transporters named SVCTs (sodium-dependent vitamin C transporters) while dehydroascorbate is transported by glucose transporters (GLUTs) (reviewed by Lykkesfeldt and Tveden-Nyborg, 2019). SVCTs (types 1 and 2) are sodium and vitamin C co-transporters and SVCT2 is more expressed in the central nervous system. On the other hand, GLUT1 and 3 are glucose transporters that have high affinity for the transport of dehydroascorbate in the central nervous system. The distribution and different location of transporters allow physiological systems to achieve different concentrations of vitamin C (reviewed by Padayatty and Levine, 2016). It has already been shown that the SVCT2 knockout condition is incompatible with life in mice that present respiratory failure and brain hemorrhage, indicating that ascorbate transport is essential in the perinatal period (Sotiriou et al., 2002).

Vitamin C is known to act as an important antioxidant agent but has other functions such as providing electrons for enzymatic reactions, participating in the formation of the myelin band, acting as an enzymatic cofactor, hormone synthesis, modulation of the glutamatergic system, regulation of gene expression, among others (May 2012; Oudemans-van Straaten et al., 2014; reviewed by Padayatty and Levine, 2016).

### 3.4 Vitamin C in the brain

Ascorbate concentrations in the brain are high. Glial cells can reach a concentration of 1 mM while neurons can reach concentrations of 10 mM. To reach the central nervous system, vitamin C must cross the blood-brain barrier. Studies show that ascorbate can reach the choroid plexus through SVCT2 reaching the cerebrospinal fluid while dehydroascorbate crosses the blood-brain barrier by GLUTs (Nualart et al., 2014). Another mechanism to maintain the high concentration of vitamin C in the central nervous system is the recycling of dehydroascorbate by astrocytes. When ascorbate is used, formation of its oxidized form occurs. Dehydroascorbate, in turn, will be taken up by astrocytic cells *via* GLUTs where it undergoes the actions of enzymes, reducing dehydroascorbate into ascorbate. When released into the extracellular environment, ascorbate can be taken up by neuronal cells *via* SVCT2 (reviewed by Covarrubias-Pinto et al., 2015). The recycling ability of vitamin C can also be observed at the blood-retinal barrier (Hosoya et al., 2004).

### 3.5 Vitamin C as a neuromodulator in the retina

Ascorbate is present in the chick retina (Portugal et al., 2009) and, in this model, can be released by reversal of SVCT2 (Figure 5). Glutamate interacts with the NMDA receptor leading to an increase in sodium in the membrane microdomain. This inversion of the electrochemical gradient of sodium generated by NMDA allows the interaction of SVCT2 with sodium ions and intracellular ascorbate and, thus, the transporters would release vitamin C instead of capturing it (Portugal et al., 2009). Another study shows that NO promotes the increase of SVCT2 levels through the activation of its canonical cGMP/PKG pathway, stimulating NFκB translocation to the nucleus and its consequent transcription. In addition, NO can increase intracellular levels of ascorbate in retinal neurons, protecting cells from damage caused by oxidative stress (Portugal et al., 2012).

Ascorbate inhibits glutamate uptake by decreasing levels of EAAT3 (excitatory amino acid transporter 3) in the cell membrane of neurons (Figure 5). Thus, there is an accumulation of extracellular glutamate and a consequent increase in the activation of glutamate receptors and downstream signaling pathways leading to CREB activation (Domith et al., 2018b). Recent studies also show that dopamine induces ascorbate release from cultured retinal neurons in a dose-dependent manner. It was observed that dopamine pulses increased the release of ascorbate without depleting its intracellular stores, an effect also dependent on EPAC2. We can conclude that the D1/cAMP/EPAC2 signaling pathway is involved in dopamine-induced acorbate release in cultured retinal cells (da Encarnação et al., 2018). Ascorbate was also shown to increase the efficiency of dopaminergic transmission by increasing the half-life of dopamine (Neal et al., 1999) and possibly potentiating the activity of the D1 receptor (Bernstein and Dillon, 2014), an effect also dependent on EPAC2.

The release of vitamin C induced by dopamine is dependent on the activation of ionotropic glutamate receptors of the AMPA/kainate type (Portugal et al., 2019). These activated receptors stimulate signaling pathways such as PI3K/AKT and MAP kinases that promote the reversion of the SVCT2 transporter (Figure 5), a mechanism that can explain how the cellular release of ascorbate occurs (Portugal et al., 2019). Adenosine, an important neuromodulator of the nervous system, also modulates vitamin C release in chick retinal cultures and this effect is dependent on the activation of adenosine A3 receptors, which associate with the SVCT2 transporter to modulate the transport of ascorbate, increasing its release into the extracellular environment (Portugal et al., 2021). This modulation of ascorbate transport may be important for neuronal homeostasis.

Data also show that ascorbate is able to modulate GABA receptors, potentiating its activity in goldfish retinas (Calero et al., 2011). In addition, clinical studies show that patients with proliferative diabetic retinopathy have decreased levels of vitamin C in the aqueous humor and vitreous humor when compared to non-diabetic patients. This may be due to the rapid depletion of vitamin C caused by increased oxidative stress (Park et al., 2019).

### 3.6 Vitamin C as a neuroprotective agent in retina

One study shows that exposure for 1 h to ultraviolet B radiation was able to cause damage to photoreceptor cells characterized by the presence of pyknotic nuclei and disappearance of the outer segments of these cells. However, damage by UVB exposure was blocked in the presence of vitamin C, possibly due to its antioxidant effects (Tokuda et al., 2007). Additionally, exposure to lead, present in cosmetics, fuel, and industrial processes, induces cellular apoptosis in rod and bipolar cells of the retina and exposure during pregnancy and lactation induces and increases apoptosis in the photoreceptor layer of rat offspring. The consumption of vitamin C during pregnancy and lactation of rats was able to reduce the damage caused by lead in the photoreceptor cells of the offspring of rats (Khordad et al., 2013). The use of topical ascorbate also exerts protective effects on corneal endothelial cells *via* PI3K/AKT in a rabbit phacoemulsification oxidative stress model (Hsueh et al., 2020).

### 3.7 Nitric oxide

Nitric oxide (NO) is an important cellular messenger produced from the amino acid L-arginine by enzymes called nitric oxide synthases (NOSs). The three NOS isoforms (the constitutive endothelial and neuronal, NOS I and II, and the inducible immunological, NOS III) are expressed in different cell types and have variable functions in physiological systems. The neuronal isoform nNOS is found in the central nervous system, present mainly in neurons (reviewed by in Luo and Zhu, 2011). Stimulation of NMDA receptors promotes an increase in the influx of intracellular calcium, and the complex calcium-calmodulin is able to activate nNOS that catalyzes the production of NO and citrulline in a stoichiometric way (Christopherson et al., 1999). NO exerts its effects by activating some signaling pathways, and the classical pathway involves activation of soluble guanylyl cyclase (sGC), cyclic GMP production and stimulation of protein kinase G (PKG or cGK). In the CNS, NO is involved in synaptic plasticity, neurotransmission, neuroprotection, sleep control, among others (reviewed by Calabrese et al., 2007). NO is also involved in the activation of the AKT pathway (reviewed by Contestabile and Ciani, 2004). Excessive NO production can lead to the formation of reactive nitrogen species and triggers cellular damage.

High levels of NO are present in the early stages of chick retina development (Ientile et al., 1996). Interestingly a NADPH diaphorase activity that corresponds to NOS was described during chick retina development and that can be stimulated by calcium ions (Paes-de-Carvalho and de Mattos, 1996). NO donors, such as SNAP, prevent cell death in purified cultures of retinal neurons from E8 embryos with involvement of the sGC/PKG and PI3K pathways, among others. In addition, SNAP treatment promoted neurite outgrowth that may be important for the development of the nervous system (Mejía-García and Paes-de-Carvalho, 2007). SNAP also reduced cell proliferation in purified cultures of retinal glial cells and in the “intact” retina model *via* a cGMP-independent pathway (Magalhães et al., 2006). Stimulation of NMDA receptors in cultures from E8 chick embryos promotes inhibition of protein synthesis leading to increased availability of the NOS substrate L-arginine. This increase in available L-arginine can be used for NO synthesis (Cossenza et al., 2006). Surprisingly, inhibition of protein synthesis is also able to promote L-arginine intracellular accumulation and a consequent increase of NO synthesis and activation of downstream signaling pathways including the AKT pathway (Cossenza et al., 2020). Experiments in intact retina of E8 embryos show that NO can modulate CREB phosphorylation, a transcription factor that plays a key role in many cellular functions (Socodato et al., 2009). Data show that stimulation of the calcium permeable AMPA receptor increases the activity of nNOS and the NO produced modulates the activity of Src kinases in cultures of E8 embryos. This effect is confirmed with the use of NO donors and a guanylyl cyclase activator (Socodato et al., 2012). Src is a kinase with important functions such as involvement in long-term potentiation (MacDonald et al., 2006). NO also increases the activity of the PI3K/AKT pathway and promotes translocation of AKT to the cell nucleus, in addition to protecting cells from a toxic stimulus induced by hydrogen peroxide (Mejía-García et al., 2013). As described in the previous section, NO modulates the expression of the ascorbate transporter SVCT2, increasing the ascorbate transport capacity *via* NF-κB and PKG (Portugal et al., 2012). The viability of neuronal cells can be controlled by NO depending on the age of the embryo. SNAP promotes cell death of retinal cells from E6 embryos while it leads to decreased apoptosis in retinas from E8 embryos. These events in this time window happen *via* sGC and cGKII. Furthermore, SNAP-induced CREB phosphorylation was increased in E8 embryos while in E6 it was decreased (Socodato et al., 2014). These data show that NO is an important modulator of neuronal survival in the developing CNS.

NO and its classical activation pathway appear to be present and exert physiological roles on retinal cells (Eldred and Blute, 2005). Studies show that NO can act both on intraocular pressure and on the retinal pathophysiology of glaucoma (reviewed by Wareham et al., 2018). nNOS is expressed by pigmented epithelium, amacrine cells, ganglion cells, and photoreceptors (Blom et al., 2012), and the NO produced by this enzyme can act as a messenger between the inner cell layers of the retina and astrocytes. The eNOS enzyme is also important in controlling vascular smooth muscle tone in the eye (reviewed by Toda and Nakanishi-Toda, 2007). Data show that NO is important for correct development of retinotectal projection in chicks (Ernst et al., 1999; Wu et al., 2001). In turtle retinas, NO enhances GABA release in horizontal cells *via* cGMP (Yu and Eldred, 2005). In developing chick retinas NO can alter the cytosolic pH of amacrine cells promoting chloride release from these cells (McMains and Gleason, 2011).

## 4 Conclusion

One of the major problems in biology is how cells in multicellular organisms build the different tissues and organs in a coordinated and precise pattern. It is generally believed that this organization depends on cellular interactions and communication. Although direct cell contacts play an important role, chemical communication between cells appears to be essential to ensure correct development. The nervous system is made up of hundreds of different neurons and glial cells that develop in a coordinated fashion forming synaptic contacts in proper circuits able to perform very complex functions. Chemical signaling in the nervous system is made primarily by neurotransmitters, neuromodulators, hormones and local mediators, and their respective receptors and signaling pathways. The retina, and specially the chick retina, is a useful model to study this molecular signaling. We have reviewed here the development of some of these signaling systems in the chick retina with emphasis in those related to cyclic AMP, such as dopamine and adenosine, and to AKT, such as glutamate and related neuromodulators as vitamin C and nitric oxide. We have also discussed the role of some of these molecules in embryonic functions such as cell survival and proliferation. We conclude that these molecules are very important for retina development and that disturbances in their function can lead to neurodegenerative disorders.
